# Unsatisfactory accuracy of recent robotic assisting system ROSA for total knee arthroplasty

**DOI:** 10.1186/s40634-022-00522-7

**Published:** 2022-08-19

**Authors:** Caleb Shin, Chelsea Crovetti, Enshuo Huo, David Lionberger

**Affiliations:** 1 Department of Orthopaedic Surgery, Houston Methodist Hospital, 6445 Main St, Houston, TX USA; 2 Texas A&M College of Medicine, Bryan, TX USA

**Keywords:** Total knee arthroplasty, Robotic surgical assistant, Computer assisted surgery, Mechanical axis, Hip-knee-ankle angle, Clinical study

## Abstract

**Purpose:**

The purpose of this study was to quantify accuracy of a recently FDA-approved robotic-assisted device.

**Methods:**

Thirty-seven patients underwent TKA with the Robotic Surgical Assistant (ROSA) by the same operating surgeon and team over the course of 3 months. Intra-operative mechanical axis measurements, composed of alpha (α), beta (β), gamma (γ), and delta (δ) angles, and the hip-knee-ankle angle (HKA) were calculated by the ROSA. Post-operative mechanical implant angles were taken from 36″ stitched post-op films and measured in the PACS imaging system. Accuracy was assessed by comparing the percentage of postoperative long length films within 2° and 3° of the ROSA intra-operative plan.

**Results:**

The ROSA system accurately calculated the HKA, α, and β angles (95% CI), but was inaccurate in calculating both γ and δ angles. Using a window of ± 3° accuracy, the HKA, α and β angles were accurate at levels of 89, 100 and 92% respectively. In contrast, the sagittal relationships were considerably less accurate at 77 and 74% for the γ and δ angles respectively. Subsequently, the proportion of cases within 2 and 3 degrees of the intra-operative plan for resection angles was considered accurate for HKA (73% within 2°, 89% within 3°), α (92% within 2°, 100% within 3°), and β (76% within 2°, 92% within 3°) angles, but considered inaccurate for γ (51% within 2°, 77% within 3°) and δ angles (57% within 2°, 74% within 3°).

**Conclusions:**

This study demonstrated that while the ROSA system seems to accurately predict coronal plane resections in TKA, it falls short in the sagittal plane. Further research in these deficiencies can provide insight into the overall efficacy of robotic assisted surgery in TKA.

**Level of Evidence:**

Level III Therapeutic Study.

## Introduction

While the use of robotic-surgical assistants in total knee arthroplasty (TKA) continues to grow, there remains a lack of evidence in whether these expensive programs are accurate in regards to implant positioning in a clinical setting. Additionally, proposed benefits of such robotic assistance such as improved patient outcomes, patient safety, and efficiency hinge on the assumption that accuracy is significantly improved compared to a TKA with traditional instrumentation. This article quantifies the accuracy of a new robotic orthopedic surgical assistant on the market and briefly reviews other literature surrounding this device to provide data for continued use and future implementation in TKA operations. The purpose of this study was to review the accuracy of a new robotic assisted surgery for TKA operations in a clinical setting.

## Materials and methods

This comparative study is approved by the Methodist IRB under PRO00024849 as of May 2021. Thirty-seven patients were enrolled in a sequential series utilizing a TKA, with the Robotic Surgical Assistant (ROSA) system (Zimmer Biomet, Warsaw, IN). Eligible patients included patients 45 years of age or older scheduled to undergo TKA with flexion contracture of less than 15 degrees**.** Patients with any medical condition or personal circumstances that would prevent completion of follow-up visits were excluded from the study. No bias or effort was contrived in recruitment of the patients. All cases were performed by the same operating surgeon and the same surgical staff without new staff trainees or resident/fellow participation. The surgeon had never used the ROSA before however had previous familiarity with the Persona TKA system (Zimmer Biomet, Warsaw, IN) and is a regular computer-assisted surgery (CAS) user for 20 years.

**Fig. 1:**
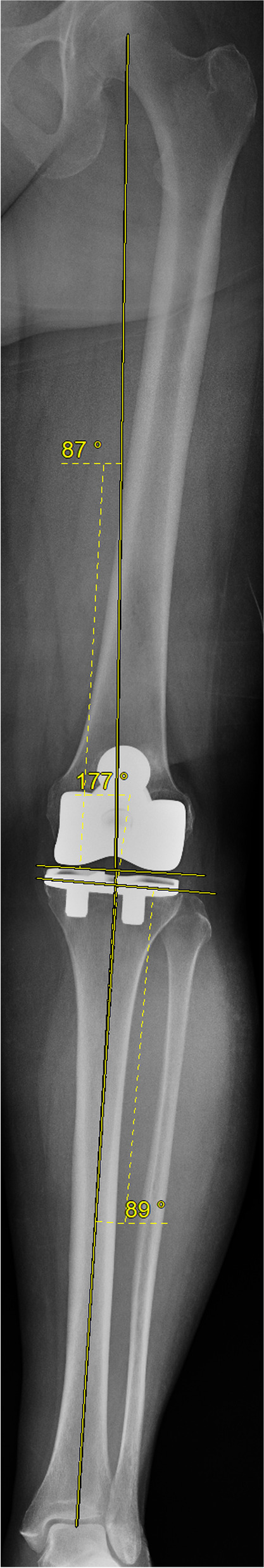
Demonstrates the alpha, and beta angle alignments on the coronal plane

**Fig. 2:**
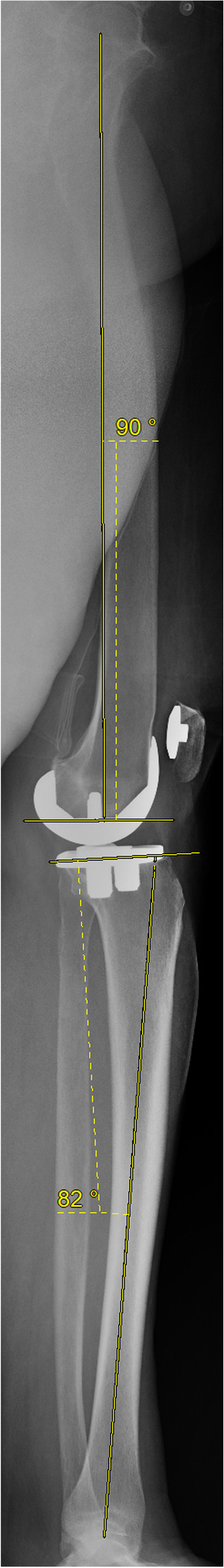
Demonstrates the gamma, and delta angle alignments on the sagittal plane

The ROSA is calibrated via waypoint acquisition marking the femoral and tibial landmarks to plan cuts. Measurements of planned surgical resections were intra-operatively planned in all cases with the ROSA software returning a “best scenario” for balancing which was subsequently recorded as the planned value for the HKA, coronal femur and tibial angle, and sagittal femoral flexion and tibial slope. After confirmation of the final values, the ROSA system intraoperatively self-positions the cutting jig to make the planned resections. The planned values are determined with the ORTHOsoft Total Knee Navigation system (Zimmer Biomet, Warsaw, IN) implemented into the ROSA. Range of motion (ROM) and fixed deformity assessment was done by an extended position of the leg held in a suspended, non-stressed manner while metrics for gap balance corrections were done under externally applied stress in 0°, 45°, and 90° flexion per the ROSA pre-op planning screen. Intraoperative sedated postoperative alignment was not used as the resultant corrected comparison, in order to not to incur confounding errors. The applied stress to the α angle, or femoral coronal angle, was defined as the resultant angle between a line drawn parallel with the femoral component condyles and the hip-knee angle (HKA) axis of the femur. The β angle, or tibial coronal angle, was the planar tangent of the tibial tray and the intercept of the center of the talus to the middle of the tray (Fig. 1). The γ angle, or the sagittal femoral angle, was defined as the point on the head, mid-distal interface of the femoral component to the femoral head tangent. The δ angle, or the tibial slope, was defined as the posterior angle between a line tangent to the tibial component to the dome of the talus (Fig. 2). Finally, the hip knee angle (HKA) was also measured. Standing 36″ stitched post-op AP and lateral films were used in the above measurements. Angles were measured by 2 blind observers using the GE Healthcare Centricity RIS/PACS 3.0 software (Chicago, IL, USA). Interobserver reliabilities were assessed using interclass correlation coefficient (ICC), where an ICC below 0.5 implies poor agreement, between 0.5 and 0.75 implies moderate agreement, between 0.75 and 0.90 implies good agreement, and above 0.90 implies excellent agreement [1]. The mean value obtained by the three reviewers were then reported as the final measure. If a radiograph was of poor quality in either the sagittal or coronal plane, the radiograph was marked for further review by an additional third observer and the average of the measured values were used.

### Statistical methods

We performed the two-sampled proportions test to compare the percentages among angles. All values are presented as mean ± standard deviation. We performed power analysis to evaluate sample sizes and effect size. Using δ of ROM as the outcome comparing ROSA and traditional surgery groups, we found a sample size of 11 in each group would suffice for an 80% power (α = 0.05). Our study sample size could detect an effect size of 1.77 degree.

## Results

A total of 37 patient's completed the follow-up for appropriate radiographic and clinical assessment follow-up. The patients were a mixed gender (27 females, 12 males), elderly (average age 67.4 ± 7.96), with an average BMI of 31.2 ± 4.99. All patients had a flexion contracture in the range of 5–10°. We summarized the absolute differences in planned and post-op for the HKA angles, α angles, β angles, γ angles, and δ angles with mean and standard deviations (Table 1). The count and percentages of patients whose planned and post-op angles were less than 2° or 3° were summarized (Table 2). Using a window of ± 3° accuracy, the HKA, α and β angles were accurate at levels of 89, 100 and 92% respectively. In contrast, the sagittal relationships were considerably less accurate at 77 and 74% for the γ and δ angles respectively. 34 out of 37 total patients (91.89%, 95%CI = [78.09–98.30]) had their planned and post-op α angle difference within 2°, and 37 (100%, 95%CI = [90.51–100.0]) within 3°. A total of 27 out of 37 patients (72.97%, 95%CI = [58.80–88.23]) had planned and post-op HKA angle difference within 2°, and 33 out of 37 (89.19%, 95%CI = [74.58–96.97]) within 3°. 28 out of 37 (75.68%, 95%CI = [58.80–88.23]) patients whose planned and post-op beta angle difference were within 2°, and 34 out of 37 (91.89%, 95%CI = [78.09–98.30]) within 3°. Sagittal accuracy was significantly worse than anterior–posterior accuracy with 18 out of 37 patients (51.43%, 95%CI = [47.46–79.79]) having planned and post-op γ (sagittal femoral) angle difference within 2°, and 27 out of 37 (77.14%, 95%CI = [71.23–95.46]) within 3°. 20 out of 37 patients (57.14%, 95%CI = [36.92–70.51]) had planned and post-op δ (tibial slope) angle difference within 2°, and 26 out of 37 (74.29%, 95%CI = [53.02–84.13]) were within 3°. The mean degrees of difference (°) in intra-operative plan versus post-op measurements for the α, β, γ, and δ angles respectively were 0.88 ± 0.71, 1.24 ± 1.06, 1.93 ± 1.03, and 2.04 ± 1.55. The number of faulty radiographs was 8, all in the sagittal plane. Interobserver reliabilities of the measurements was moderate with an ICC of 0.741 for HKA measurements, 0.729 for the alpha angle measurements, 0.558 for the beta angle measurements, 0.782 for the gamma measurements, and 0.635 for the delta angle measurements. In the series of patients there was no trend nor statistical significance in terms of accuracy of either improvement over time with the familiarity of the surgeon accomplishing better results nor with disparity or variance in consideration of the degree of deformity.

**Table 1:** Accuracy of ROSA to reproduce intra-operative plan for bone resection angles

	**Mean |∆|± SD [95% CI]**	**Min–Max**
**Alpha**	**0.88 ± 0.71 [0.65–1.12]**	**0.00–2.90**
**Beta**	**1.24 ± 1.06 [0.89–1.60]**	**0.00–3.90**
**Gamma**	**1.93 ± 1.03 [1.58–2.29**	**0.30–4.00**
**Delta**	**2.04 ± 1.55 [1.51–2.57]**	**0.10–6.00**

**Table 2:** Proportion of Cases within 2 and 3° of the intra-operative plan for bone resection angles

N (%)	Post-OP vs. Planned < 2º	[Percentage 95% CI]	Post-OP vs. Planned < 3º	[Percentage 95% CI]
**HKA**	**27.00 (72.97)**	**[58.80–88.23]**	**33.00 (89.19)**	**[74.58–96.97]**
**Alpha**	**34.00 (91.89)**	**[78.09–98.30]**	**37.00 (100.00)**	**[90.51–100.0]**
**Beta**	**28.00 (75.68)**	**[58.80–88.23]**	**34.00 (91.89)**	**[78.09–98.30]**
**Gamma**	**18.00 (51.43)**	**[47.46–79.79]**	**27.00 (77.14)**	**[71.23–95.46]**
**Delta**	**20.00 (57.14)**	**[36.92–70.51]**	**26.00 (74.29)**	**[53.02–84.13]**

## Discussion

Implementation of computer of technology in TKA is a constantly evolving process as CAS continues to develop in an effort to optimize surgical outcomes, patient safety, efficiency, and cost effectiveness [2–9]. Recent literature suggest that the addition of such mechanical assistance be it through haptic or passive guidance with robotic technology may improve these aspects of CAS. There has also been suggestion of reduced periarticular soft tissue damage [2, 3, 9], reduced postoperative pain, reduced bleeding, decreased opiate requirement, shorter length of stay, and greater patient satisfaction [2, 3, 9]. The ROSA has been reported to provide some of these improvements [10, 11]. Through a series of waypoint acquisitions to orient the computer to either image based from preoperative radiographs or non-image based, intraoperative stress data can be used to preplan resections. Using this surgeon driven selection of desired implant placement, the ROSA system can be programmed to place appropriate cutting guides in position. It does not however, aid in the guidance of the actual resection through haptic limitations of blade, bure, or a drill placement. As higher costs and time expenditure are associated with using CAS, orthopedic surgeons must validate worthy improvement to justify adoption of new technology [3], though recent literature suggest that these costs may be offset by cost savings from shorter hospital stays, reduced postoperative costs, and reduced healthcare resource utilization [2, 3, 9]. With proposed improvements in surgical accuracy and benefits in outcomes and cost efficacy, robotic-assisted surgery continues to be a worthwhile endeavor to explore. Additionally, wider adoption and experience with the technology may continue to improve outcomes and cost benefits associated with robotic surgery. The purpose of this study was to assess one specific aspect of accuracy of ROSA by comparing the desired mechanical axis calculated intraoperatively by the ROSA computer versus the actual mechanical axis measured post-operatively with long-length knee radiographs.

This study calls into question strictly the accuracy of a novel robotic technology, ROSA, and provides insight into how it can be further improved or adjusted to justify the costs associated with it. In the anterior–posterior resection planes (HKA, α, and β), accuracy was exceptional. 91.89% of patients were correct to within 2° and 100% within 3° of the α angle. 72.97% of patients were correct to within 2° whereas 89.19% were correct to within 3° of the HKA angle., and finally 75.68% were within 2° and 91.89% within 3° on the β angle. However, the angles measured in the sagittal planes did not measure as consistently compared to the coronal plane resections. 51.43% of patients were within 2° on the γ angle, or sagittal femoral angle, whereas 77.14% of patients were within 3° of correct alignment. 57.14% of patients were correct to within 2° on the δ angle, or tibial slope and 74.29% were within 3° of correct alignment. These results differ from previous studies by Seidenstein et al. [11], who reported 92.9, 100, 71.4, 100, and 100% of planned HKA, α, β, δ, and γ knee angle respectively within 2° of the post-op angles, and 100%, 92.9%, 100%, 100%, 100% within 3° respectively. Another similar study by Parratte et al. [12] reported 97%, 100%, 100%, 87%, 100% of planned HKA, α, β, δ, and γ within 2° of the post-op angles, and 100%, 100%, 100%, 97%, 100% within 3°. These previous studies were performed on cadavers using the same CAS system, ORTHOsoft Total Knee Navigation system (Zimmer Biomet, Warsaw, IN). The ORTHOsoft Total Knee Navigation system is an infrared camera-based imageless navigation system that has reported accuracy of less than 1 mm and 1° [1]. Given this accuracy and our results in the sagittal plane, there appears to be a disconnect in the transfer of data from the navigation system to the cuts. Whether this disconnect is based of the navigation system itself, the act of calibrating anatomic landmarks, or the movement of the cutting jig to the cutting surface of the knee, is difficult to ascertain. However, further research is required to evaluate these variables and larger studies are required to determine the repeatability of these results.

The HKA or mechanical angle is more often reported in outcomes studies as the litmus test for accuracy and likewise survivability and functional outcomes. Inaccuracies derived on the sagittal plane may likely be more tolerated. From a cosmetic standpoint, such as a hyperflexed femoral component or altered tibial slope resulting in a relatively normal function in short-term clinical outcomes. However, a singular malpositioned femoral or tibial component results in imbalance flexion/extension gap, resulting in motion loss, midflexion instability, and ultimately early failure. While the ROSA is an accurate option, with regards to the mechanical/HKA, α, and β angles, it fell short in accuracy which was evidenced by the decrease accuracy percentages of the δ and γ knee angles. Other studies on CAS system accuracies have been described and shown that they have improved accuracy in these planes using both CT, and CT free systems [13–15].

There were several short falls and limits of this study. The first limitation is the use of radiographs which fall short in capturing rotation abnormalities as it is only a 2-dimensional view to measure a 3-dimensional construct. Additionally, although our technicians are thoroughly and skillfully trained, the x-ray technician taking each radiograph was not controlled, leaving room for variability in the results. The gold standard for accuracy is spiral CT imagery which is extremely expensive, difficult to attain, and more importantly exposes the patients to excessive radiation. That being said, the bulk of studies still rely on 36 inch imagery which given the comparison and literature seems to be a fair assessment of this study against those of others.

Additionally, this study was a sequence non-rehearsed start-up study with the senior author having had 20 years of experience in computer-assisted surgery (CAS). However, the surgeon was not familiar with the ROSA system when he started in an attempt to get a real world experience of the typical surgical expected outcomes rather than correcting and making up for deficiencies and technical expertise and skills for instance, we noted during the placement of pins on the ROSA system a ‘pin drift’ occurs as a result of the potential inefficiencies in the haptics installed in the ROSA system to hold it steady. Even in the best of circumstances, in hard bone with geometric curvatures, a pin skid can potentially occur unbeknownst to the artificial intelligence and haptic technology of the ROSA system. The haptics that control the rigidity of the arm based off of the servos which are electronically controlled stabilizers did not appear to have complete resistance to this deficiency. This error then follows throughout the entire procedure and in the end reflects itself at the end in the inaccuracies of the proposed planned angles of the knee and what was actually obtained in post-operative radiographic imaging. While in the latter series of cases this pin skid was attempted to be corrected, there did not appear to be a significant difference in the accuracy obtained in the later cases over those of the first. Therefore the significance of this while trying to reconcile inaccuracies may or may not be the root cause of this downfall. Finally, 36 inch long films taken which are the standard for accuracy have their own inherent limits. While utilizing the best of radiographic techniques, rotation and inconsistencies exist in any type of radiographic follow-up inherent with this type of measurement. The fact that a number of patients were used as well as this study being compared to previous studies using the same techniques will tend to normalize this variance.

## Conclusion

This study shows the ROSA as exceptionally accurate in the coronal plane yet marginally acceptable in the sagittal. This suggests that more research and development may be required to refine the deficiencies and short falls in the sagittal plane of the δ and γ implant positionings. Given the enhanced cost linked to the additional drains on operative time and surgeon disruption of normal workflow, there must be accountability to justify such technological inclusions. Given the discrepancies in accuracy reproduction demonstrated in the study, it implores industry to refine and, or correct these technologies such that they become more valued in terms of the precision they offer. We believe these outcomes are clinically relevant to practicing Orthopaedic surgeons who seek modalities to improve patient outcomes while not at the expense of significantly decreased productivity and output. Further studies must be conducted to determine whether the deficiency in accuracy translates to significant functional outcomes in patients with computer assisted surgery.
